# Aqueous Fraction of *Beta vulgaris* Ameliorates Hyperglycemia in Diabetic Mice due to Enhanced Glucose Stimulated Insulin Secretion, Mediated by Acetylcholine and GLP-1, and Elevated Glucose Uptake via Increased Membrane Bound GLUT4 Transporters

**DOI:** 10.1371/journal.pone.0116546

**Published:** 2015-02-03

**Authors:** Ashraf Ul Kabir, Mehdi Bin Samad, Arif Ahmed, Mohammad Rajib Jahan, Farjana Akhter, Jinat Tasnim, S. M. Nageeb Hasan, Sania Sarker Sayfe, J. M. A. Hannan

**Affiliations:** 1 Department of Pharmaceutical Sciences, North South University, Dhaka, Bangladesh; 2 Pharmacy Discipline, Life Science School, Khulna University, Khulna, Bangladesh; Consiglio Nazionale delle Ricerche, ITALY

## Abstract

**Background:**

The study was designed to investigate the probable mechanisms of anti-hyperglycemic activity of *B. Vulgaris*.

**Methodology/Principal Findings:**

Aqueous fraction of *B. Vulgaris* extract was the only active fraction (50mg/kg). Plasma insulin level was found to be the highest at 30 mins after *B. Vulgaris* administration at a dose of 200mg/kg. *B. Vulgaris* treated mice were also assayed for plasma Acetylcholine, Glucagon Like Peptide-1 (GLP-1), Gastric Inhibitory Peptide (GIP), Vasoactive Intestinal Peptide, Pituitary Adenylate Cyclase-Activating Peptide (PACAP), Insulin Like Growth Factor-1 (IGF-1), Pancreatic Polypeptides (PP), and Somatostatin, along with the corresponding insulin levels. Plasma Acetylcholine and GLP-1 significantly increased in *B. Vulgaris* treated animals and were further studied. Pharmacological enhancers, inhibitors, and antagonists of Acetylcholine and GLP-1 were also administered to the test animals, and corresponding insulin levels were measured. These studies confirmed the role of acetylcholine and GLP-1 in enhanced insulin secretion (p<0.05). Principal signaling molecules were quantified in isolated mice islets for the respective pathways to elucidate their activities. Elevated concentrations of Acetylcholine and GLP-1 in *B. Vulgaris* treated mice were found to be sufficient to activate the respective pathways for insulin secretion (p<0.05). The amount of membrane bound GLUT1 and GLUT4 transporters were quantified and the subsequent glucose uptake and glycogen synthesis were assayed. We showed that levels of membrane bound GLUT4 transporters, glucose-6-phosphate in skeletal myocytes, activity of glycogen synthase, and level of glycogen deposited in the skeletal muscles all increased (p<0.05).

**Conclusion:**

Findings of the present study clearly prove the role of Acetylcholine and GLP-1 in the Insulin secreting activity of *B. Vulgaris*. Increased glucose uptake in the skeletal muscles and subsequent glycogen synthesis may also play a part in the anti-hyperglycemic activity of *B. Vulgaris*.

## Introduction

*Beta vulgaris* subsp. Cicla, also known as Chard, is a leafy green vegetable, with a red stalk and is widely consumed throughout the world. It has been traditionally used as an anti-hyperglycemic nutritional supplement in Turkey[1]. Nutrition therapy is an important part of diabetes self-management education. The primary objective of diabetes self-management education has been to reduce the incidence of various diabetic complications, to minimize morbidity, and to reduce overall treatment cost[2]. However, concrete evidence regarding this is sparse, and misconceptions about this method of diabetes management, amongst patients, are rife[3]. Studies conducted by Ş Bolken et al (2000), have shown an increase in the number of β-cells in diabetic rats on B. vulgaris administration. Other significant observations made in this study were an increase in number of secretory granules, low density granules and hypertrophic Golgi bodies. Overall, the study predicted possible β-cell regenerating capability of *B. vulgaris*[4]. Anti-hyperglycemic activity of *B. vulgaris* was demonstrated on diabetic rabbits as well[1]. Histological examinations, assay of serum urea, and creatinine of *B. vulgaris* treated diabetic rats also showed significant nephro-protective activity of this plant extract[5]. Biochemical studies conducted on hepatic enzyme functions ascertained significant protection imparted upon the liver of diabetic rats[4]. Additionally, investigators have shown that *B. vulgaris* might help to retard the onset of various ocular complications, commonplace in diabetic patients[6]. *B. vulgaris* also demonstrated to have a highly promising anti-oxidative effect in studies on diabetic rats. The study showed a reversal in the increased lipid peroxidation and lowered glutathione levels in the aorta and cardiac tissues of diabetic rats[7]. Other studies have corroborated this finding by investigating the anti-oxidant properties of this plant[8]. From this body of available literatures, we have taken a particular interest in the works of ŞBolken et al (2000). The authors of this paper have concluded potential β-cell regeneration behind the anti-hyperglycemic activity of *B. vulgaris*. However, they fell short of providing any stand on the increased number of secretory vesicles seen in the extract treated group. It is widely known that plant extracts often contain a large amount of carbohydrates, which are eventually digested to glucose, hence, might be the principal stimulus for insulin secretion. Therefore, our study has been designed to identify the non-glucose stimulants that might be responsible for any enhancement in level of insulin secretions observed. For this study, we have chosen a total of 10 hormones, based on their established significance in insulin secretion. Epinephrine, Noreinephrine, Acetylcholine, Gastric Inhibitory Peptide (GIP), Vasoactive Intestinal Peptide (VIP), Glucagon like Peptide-1 (GLP-1), Pituitary Adenylate Cyclase-activating Peptide (PACAP), Insulin Like Growth Factor-1 (IGF-1), Pancreatic Polypeptides (PP) and Somatostatin were assayed in conjunction with the corresponding in-vivo plasma insulin levels. Among all the above hormones, plasma acetylcholine and GLP-1 levels were found to be elevated in B. vulgaris administered mice. To fully confirm the role of these two hormones in the extract influenced insulin release, we administered *B. vulgaris* in conjunction with the respective pharmacological inhibitors, enhancers and antagonists of acetylcholine and GLP-1 to manipulate their plasma levels or to eliminate their effect. The resultant plasma insulin was observed after each intervention. We replicated the same in-vivo concentration of Acetylcholine and GLP-1 in-vitroand exposed the mice islets, isolated from *B. vulgaris* pre-treated mice, to observe whether the concentration of Acetylcholine and GLP-1 observed in-vivo are capable of activating the insulin release cascade in-vitro. The second part of this paper entailed observing the glucose uptake and determining fate of this glucose. Membrane bound GLUT-1, GLUT-4 transporters and glucose-6-phosphate were quantified. Hexokinase II activity and glycogen synthase activity were determined along with the glycogen deposited in the skeletal muscles.

## Material and Methods

### 1. Plant Collection and Processing

*B. vulgaris* (whole plant) was procured from University Ayurvedic Research Centre (UARC), Jahangirnagar University, Dhaka, Bangladesh. Identity of the plant material was confirmed by a botanist, and a voucher specimen was deposited at the National Herbarium at Mirpur, Dhaka, Bangladesh. The plant materials were rinsed under running tap water, air-dried in an oven at 40°C, and milled into a fine powder. 500gm of the powder was added to 5L ethanol, and mixed with the help of an orbital shaker (550rpm for 48hrs). The plant-ethanol mixture was filtered using fine a muslin to eliminate the coarse insoluble particles. Centrifugation (1500rpm for 10mins) helped to sediment the finer particles. The supernatant was pipetted out and re-filtered using Whatman filterpaper. The filtrate was concentrated usingSoxhlet apparatus (ElectrothermalSoxhlet extractor, UK). This concentrate was left in a refrigerator for 7days to remove further moisture, converting it into a gummy mass. This mass was then fractionated following the method described by Hannan et al. [9]. The ethanol extract was subsequently partitioned between n-hexane and water. n-Hexane fraction was separated and freeze dried. The water layer was collected for further processing. The layer was further partitioned first by chloroform, then ethyl acetate and finally by 1-butanol. The organic fractions were separated and individually freeze dried every time. The residual aqueous fraction was condensed by rotary evaporator and finally freeze-dried. Freeze drying took place at −55°C to obtain a fine powder. The powdered extracts of all the different fractions were kept in zipper bags along with silica gel sachets (desiccant) until further use.

### 2. Animal Handling

Type 2 diabetic mice (db/db type) were procured from Harlan Laboratories (USA) and raised in the animal house of the Department of Pharmaceutical Sciences, North South University. The mice weighted about 27±2g (24.7–28.9g). All test animals were kept in the North South University Animal house at an ambient temperature of 22±5°C and humidity of 50–70%. 12hrs day-night cycle was maintained to avoid fluctuations in the circadian rhythm. Standard pellets and filtered drinking water were made available to the test animals, ad libitum, throughout the experiment, apart from the period of fasting prior to certain tests. During fasting, only water was given. During most of the experimental period, the mice were singly kept in translucent plastic cages, with sterilized wood shavings provided as bedding. Animals undergoing fasting were placed in grilled bottomed cages, with no bedding, to prevent corpophagy. The mice were tagged with an I.D. number, which was fed into a computer program. This allocated 10 mice to each group, at random. The caretaker of the animal house was responsible for administering the test compounds to the animals. The groups were randomly assigned numbers and the investigators were unaware of the medication status of the animals in each box till data analysis was completed. Many previous studies on *Beta Vulgaris* were carried out at doses up to 2000mg/kg, and was non-toxic at that dose[1,4,6]. In our chronic in-vivo screening (fed once daily for 8weeks), only the aqueous fraction showed anti-hyperglycemic activity and was chosen for this study. The minimum dose required for this activity was 50mg/kg (unpublished data). Multiple factors (100mg/kg, 200mg/kg and 400mg/kg) of this dose were used for a dose dependent study. Since both 200mg/kg and 400mg/kg doses were found to show activity, we chose 200mg/kg as the dose for our mechanistic study. This activity reached the peak at 30mins, hence was determined as the optimum time point for our subsequent hormonal assays. The procedure was re-conducted with a fresh group of mice after 8weeks of *B. Vulgaris* administration, after which, various blood hormones and the corresponding insulin levels were determined. Acetylcholine and GLP-1 levels were found to increase with a corresponding rise in plasma insulin levels and were chosen for our subsequent mechanistic studies. All other hormones were excluded from our study beyond this point. A fresh batch of mice were re-administered *B. Vulgaris* for another 8weeks, at the end of which, the mice were administered a final dose of *B. Vulgaris* (200mg/kg) along with pharmacological inhibitors, enhancers or antagonists of Acetylcholine and GLP-1. Plasma levels of these two hormones were then determined along with plasma insulin levels. Simultaneously, another batch of mice was treated with *B. Vulgaris* along with a control group for a period of 8weeks, after which the mice were sacrificed and their islets were harvested.

### 3. Ethics Statement

The designed experimental protocol was approved by the Ethics Committee on Animal Research, North South University. It was designed following the “Revised guide for the care and use of laboratory animals by American Physiological Society”[10]. All animals were treated humanely throughout the course of the experiments and maximum care was taken to minimize pain of the experimental animals. All surgical procedures were carried under sodium pentobarbitone anesthesia. Maintenance of the anesthesia was continuously monitored at 15mins interval by “toe pinch”. At the end of the experiment the animals were euthanized using an overdose of xylazine and ketamine.

### 4. Acute effect of aqueous fraction of *B. vulgaris* on glucose homeostasis and insulin secretion (OGTT Studies)

Oral Glucose Tolerance Test (OGTT) was performed following 8weeks of *B. vulgaris* administration, as described previously[11]. Briefly, after a designated fasting period, animals were anesthetized with an intra-peritoneal injection (100 mg/kg) of pentobarbitone sodium (Therapon, Burwood, Victoria, Australia), and a silastic catheter filled with heparinized saline (20 U/ml) was inserted into the left carotid artery. The mice also underwent tracheotomy to facilitate breathing. A bolus of glucose was delivered into the stomach by a gavage needle (20-gauge, 38 mm long curved, with a 21/4 mm ball end; Able Scientific, Canning Vale, Western Australia, Australia), and 200 μl of blood was sampled at 0, 30, 60, 90, and 120mins for plasma glucose and insulin analyses. Blood was immediately centrifuged and the plasma was separated and stored at −20°C until assayed. The red blood cells were re-suspended in an equal volume of heparinized saline and re-infused into the animal via the carotid artery prior to the collection of the next blood sample to prevent anemic shock. Blood glucose levels were analyzed by GOD-PAP method[12] (glucose kit, Randox, UK) and plasma insulin levels were determined using Mice Insulin ELISA Kit (Crystal Chem, USA)

### 5. Blood collection, plasma preparation and biochemical analysis of various plasma parameters

Following the OGTT studies, the time point of maximum insulin secretion was identified and the levels of various endogenous insulin secreting or inhibiting hormones were measured at this time point. In the current study, plasma insulin was found to be the highest at 30mins after the extract administration and hence was chosen to be the time point of measurement for further in-vivo assays. Plasma insulin levels were re-measured at this point to get a co-relation of insulin with the other hormonal levels. Those hormones, which did not show a rise in levels, on *B. Vulgaris* treatment, were not studied further. While those hormones, which exhibited a rise in plasma levels, in correspondence with insulin increase, were further analyzed using a plethora of positive and negative controls, pharmacological enhancers, inhibitors or antagonists of the hormones. This intervention helped us fully confirm the role of those hormones and allowed us to paint a mechanistic picture of the anti-hyperglycemic activity of *B. vulgaris*. Quantification of all biochemical parameters in this section was done using Colorimetric, ELISA or EIA methods following the manufacturer’s instruction accompanying the kit. Plasma Insulin levels were measured using Ultra-sensitive mice insulin ELISA Kit (Crystal Chem, USA). Plasma Acetylcholine levels were determined by a colorimetric Choline/Acetylcholine Quantification Kit (Abcam, USA). Plasma epinephrine was measured using Epinephrine ELISA Kit (Abnova, Taiwan). Plasma norepinephrine was assayed using the norepinephrine ELISA assay kit (Eagle Biosciences Inc. USA). Plasma GIP was assayed using the Rat/mouse GIP ELISA assay kit (Total) (EMD Millipore, USA). Plasma VIP was assayed using the VIP ELISA assay kit (USCN Life Sciences Inc, China). Plasma PACAP was assayed using the mouse PACAP ELISA assay kit (MyBioSourceInc. USA). Plasma IFG-1 was assayed using the mouse IGF-1 ELISA assay kit (Sigma Aldrich, USA). Plasma Pancreatic Polypeptide was assayed using the mouse Pancreatic Polypeptide ELISA assay kit (MyBioSourceInc. USA). Plasma Somatostatin was assayed using the Somatostatin EIA assay kit (Phoenix Pharmaceutical Inc. USA).

### 6. Analysis for confirming the role of *B. vulgaris* mediated increase in acetylcholine on enhanced insulin secretion after *B. vulgaris* treatment

8weeks *B. vulgaris* fed diabetic mice were taken and fasted for 24 hours before the following procedure. The mice were administered aqueous fraction of *B.vulgaris* at a dose of 200 mg/kg alone or with different acetylcholine antagonists, inhibitors, and enhancer. These pharmacological agents were injected intravenously 30mins before *B. Vulgaris* administration. A glucose load (2.5 g/kg) along with *B. vulgaris* (200mg/kg) was then administered. The untreated control group received only vehicle (water; 10 ml/kg) at all points of the experiment. Atropine, a general muscarinic receptor antagonist, was administered iv at a dose of 0.1 mg/kg to observe the role of increased acetylcholine binding in insulin release. Hemicholinium-3 (HCM-3), a choline uptake blocker, was injected iv at a dose of 0.1 mg/kg; thus inhibiting the rate limiting step of acetylcholine synthesis, lowering the plasma acetylcholine level. Vesamicol (VML), an inhibitor of vesicular acetylcholine transport, was administered iv at a dose of 3.5 mg/kg, consequently reducing acetylcholine release and plasma acetylcholine levels. Physostigmine (PTM), a reversible cholinesterase inhibitor was injected iv at a dose of 0.5 mg/kg, thereby inhibiting breakdown of acetylcholine and increasing its level. The receptor blocker and modulators of acetylcholine, along with the corresponding insulin levels help us fully ascertain the role of enhanced acetylcholine by *B. Vulgaris* treatment in insulin secretion.

### 7. Analysis for confirming the role of *B. vulgaris* mediated increase in GLP-1 on enhanced insulin secretion after *B. vulgaris* treatment

GLP-1 was determined from the same plasma sample as before (30mins) by using GLP-1 EIA Kit (Sigma-Aldrich, USA). To further confirm the role of GLP-1 in mediation of *B. Vulgaris* induced insulin release, we employed Exendin 9–39 (Ext9), a potent GLP-1 receptor antagonist, at a dose 300 pmol/kg/min. Ext9 was administered through a femoral vein catheter continuously for 30mins, while the animal subjects were kept under sodium pentobarbital anaesthesia. Saxagliptin (SxLn), an inhibitor of dipeptidyl peptidase-4 (DPP-4), at a dose of 10μmol/l administered orally 4 h before the glucose load and *B. Vulgaris* treatment.

### 8. Isolated islet preparation

Mice pancreatic islets were isolated by collagenase digestion as previously described by Li and colleagues [13]. A digesting solution was prepared by dissolving Collagenase XI in Hank’s Balanced Salt Solution (1000U/ml). The mouse was fully anesthetized and sacrificed by cervical dislocation. The location of the ampulla was clamped with surgical clamps on the duodenum wall to stop the bile pathway to the duodenum. The pancreas was distended by injecting 3mL of the digesting solution via the common bile duct. It was then removed and placed in a in a 50ml vial containing 2mL of the digesting solution. The vial was placed in a water bath at 35.7°C for 15mins and briefly shaken two to three times, by hand, during the incubation period. At the end of the incubation period, the vials were hand-shaken to disrupt the pancreas until the suspension was completely homogenous. The digestion reaction was terminated by putting the vial on ice and by adding 25mL CaCl_2_ (1mM) supplemented HBS Buffer (CAHBS). It was then centrifuged at 290g for 30s at 4°C and the supernatant was discarded. The pellet was re-suspended in 20mL ice-cold CAHBS. This process was repeated for a second time to fully stop the digestion and the resulting pellet was re-suspended with 15mL of CAHBS. The re-suspended pellet was poured through a pre-wetted 70μm cell strainer. The vials were washed with 20mL of CAHBS to ensure that negligible numbers of islets clung to the vial wall. Another 25mL of the CAHBS solution was poured through the strainer. The strainer was inverted over a sterile petri dish and thoroughly rinsed with the nutrient medium (glutamine-L 20mM, penicillin 100U/mL, streptomycin 100uL/mL, 10%FSB buffer in RPMI 1640 medium) to capture the islets. Islets were hand-picked using a wide-tipped pipette, counted and placed in 5% CO_2_ incubator at 37°C.

### 9. Analysis of pathway activation of Acetylcholine mediated insulin secretion

Acetylcholine mediated insulin secretory pathway recruits a myriad of cell signaling molecules, the most prominent of which are Phospholipase C (PLC), Diaceylglycerol (DAG) and Protein Kinase C-alpha (PKC-α)[14]. In the current study, we incubated the previously isolated islets (25 islets per group) from *B. Vulgaris* treated and untreated mice with observed concentrations of Acetylcholinein-vivo (Treated: 198 pg/ml, Untreated: 123 pg/ml). A third batch of islets from the *B. Vulgaris* treated group was prepared, which was incubated with Atropine along with the in-vivo concentration of acetylcholine as previously found (BV+Atropine treated: 189 pg/ml). The isolated islets were incubated in the same nutrient medium as used before (glutamine-L 20mM, penicillin 100U/mL, streptomycin 100uL/mL, 10%FSB buffer in RPMI 1640 medium). The islets were incubated inside a CO_2_ incubator at 5% CO_2_ concentration in a temperature of 37°C for 30mins. Viability and uniformity of function of each batch of isolated islets were ensured by determining the insulin secretion prior to the experiment (2.9±0.21 ng/mg islet protein). At the end of the incubation period, the islets were homogenized over ice using a glass hand-held homogenizer. The homogenates were immediately assayed for PLC activity, PKC-α activity and DAG levels using commercially available kits. PLC activity was determined using EnzChek Direct Phospholipase C Assay Kit (Invitrogen, USA). DAG levels were quantified using ELISA Kit for Mouse DAG (Uscn Life Science Inc, China). Specific PKC-α activity was assayed using Protein Kinase C assay kit. PKC-α specific substratewas used for in assay (Panvera, USA). The concurrent insulin secretion in the medium was measured using Ultra Sensitive Mouse Insulin ELISA Kit (CrystalChem, USA).

### 10. Analysis of pathway activation of GLP-1 mediated insulin secretion

GLP-1 mediated insulin secretion employs a number of key signaling molecules, the most significant of which are Protein Kinase A (PKA) and cyclic Adenosine Monophosphate (cAMP)[15]. In the current study, we incubated the previously isolated islets (25 islets per group) from treated and untreated mice with GLP-1 concentrations observed in-vivo (Treated: 21.4 pmol/l, Untreated: 14.7 pmol/l respectively). Third batch of islets from the treatment group was prepared, which was incubated withExtendin(9–39)(2μmol/l), along with the in-vivo concentration of GLP-1 as previously found (BV+Extendin: 21.5 pmol/l). The isolated islets were incubated in the same nutrient medium as previously used (glutamine-L 20mM, penicillin 100U/mL, streptomycin 100uL/mL, 10%FSB buffer in RPMI 1640 medium). The islets were incubated inside a CO_2_ incubator at 5% CO_2_ concentration, at a temperature of 37°C for 30mins. Viability and uniformity of function of each batch of isolated islets was ensured by determining the insulin secretion prior to the experiment (2.97±0.17 ng/mg islet protein). At the end of the exposure period, the isolated islets were homogenized over ice using a glass hand-held homogenizer. The homogenates were immediate assayed for PKA activity, cAMP level, and Insulin levels using commercially available kits. PKA activity was determined using PKA Activity Assay kit (Arbor Assays, USA). cAMP levels were determined using cAMP Direct Immunoassay Kit (abcam, USA). The concurrent insulin secretion in the medium was measured using Ultra Sensitive Mouse Insulin ELISA Kit (CrystalChem, USA).

### 11. Collection, homogenization and subsequent preparation of total cell membrane fraction from mice skeletal myocytes

At the end of the 8weeks study period, the final dose of *B. vulgaris* was administered. The test animals were sacrificed 30mins later by cervical dislocation, skeletal muscle from the hind limb was removed and rapidly dissected free of connective tissues. Muscle from the individual mice was placed in Tris buffer (pH 7.4, 20mM Tris-base, 0.05M sucrose, 0.1mM EDTA, 5μg/mL lupeptin, 5μg/mL aprotinin, 1μ/mL pepstatin, and 400μM phenylmethanesulfonyl fluoride). 1g of the tissue was placed in 5mL of the Tris buffer. Total membrane fraction was prepared following methods described by Klip et al.[16] with minor modifications as per Baron and colleagues[17]. In short, the dissected muscle tissue was finely homogenized using a glass homogenizer (five 5sec bursts at a setting of 5 and then with 10 up-and-down strokes of a teflon pestle). Aliquots of the homogenate were immediately assayed for Hexokinase II activity, glucose-6-phosphate, Glycogen synthase activity, and muscle glycogen content, while the remainder was further processed to obtain the membrane fraction. The homogenate was centrifuged at 1000g for 10mins and the supernatant was collected. The resultant pellet was re-suspended in Tris buffer and re-homogenized using the same instrument and method, as previously described, and re-centrifuged at 1000g for further 10mins. The resultant pellet was discarded and the second supernatant wasadded tothe first supernatant, thoroughly handshaken for 1mins and centrifuged at 9000g for 10mins. The resultant supernatant derived from this process was centrifuged at 190000g for 1hr. This was discarded and the pellet was re-suspended which made up the total membrane fraction. The membrane was kept at −80°C until further analysis.

### 12. Effects of *B. vulgaris* on GLUT1 and GLUT4 transporter content in membranes of skeletal myocytes

Pre-prepared membrane fraction of mice skeletal myocytes were assayed to determine the amount of membrane docked GLUT1 and GLUT4 receptors in the skeletal myocytes. Membrane bound GLUT1 and GLUT4 transporters were quantified using ELISA kit procured from UScn Life Science Inc. (USA) following the manufacturer’s instructions.

### 13. Effects of *B. vulgaris* on Hexokinase II activity in skeletal myocytes

Hexokinase II activity was determined from the muscle homogenate by the Hexokinase II Colorimetric Assay (Sigma Aldrich, USA). The activity was determined by a coupled enzyme assay, where glucose is converted to glucose-6-phosphate by Hexokinase II. This is subsequently oxidized by glucose-6-phosphate dehydrogenase to form NADH. The NADH formed, reduces a colorless probe forming a colorimetric product proportional to the Hexokinase II activity present whose absorption takes place at 450nm.

### 14. Effects of *B. vulgaris* on Glucose-6-phosphate content in skeletal myocytes

Glucose-6-phosphate from skeletal muscle homogenate was quantified in the muscle myocytes using a colorimetric assay kit (Abcam, USA). In this assay, glucose-6-phosphate was oxidized with the generation of a product which converts a nearly colorless probe to a colorimetrically active product which shows an absorbance at 450 nm.

### 15. Effects of *B. vulgaris* on enzyme glycogen synthase activity

The muscle homogenate was diluted 300 times and the glycogen synthase activity was assayed following a method described by Danfoth et al.[18]. Briefly, the reaction mixture was comprised of Tris Buffer 50mM, MgCl_2_ 12.5mm, EDTA 1mM, mercaptoeathanol 2.5 mM, UDP-D-gucose 0.75, and 1% glycogen. The assay was carried out in the presence of 0.1mM and 10mM glucose-6-phosphate. The reaction was quenched by heating in a thermostatically controlled boiling water bath for 70secs. This helped to denature the proteins which was subsequently removed by centrifugation at 400g. The supernatant was collected and assayed for UDP. This was done by reacting UDP with phosphenol pyruvate in the presence of the enzyme pyruvate kinase. The pyruvate formed as a result was made to react with DPNH in presence of the enzyme lactate dehydrogenase. DPNH gradually disappeared which was specrophotometrically followed. Results were expressed as nmol/min/mg of extract protein.

### 16. Effects of *B. vulgaris* on muscular glycogen content

Around 15mg muscle tissue was collected from the left hind leg of the mice. 10 mg of the muscle tissue was homogenized along with 200 μl of H_2_O on ice. The homogenate was boiled for 5mins to inactivate all enzymes.The boiled samples were spinned at 13000 rpm for 5mins to remove insoluble materials; the supernatant was then assayed for glycogen using a colorimetric Glycogen assay kit (Abcam, USA) as per the instruction booklet. The background glucose was measured in separate wells, before addition of the hydrolytic buffer and subtracted from the final value.

### 17. Statistical analysis

Statistical tests were conducted using Statistical Package for Social Science Software (SPSS) ver. 20 (IBM, Inc., Chicago, IL, USA). Results were presented as mean±SD. Data from experimental groups were compared using unpaired Student’s t test and the Mann–Whitney U test, as required. Experiments where data was collected at several time intervals, were analyzed using repeated measures ANOVA followed by Bonferroni adjustment ensuring an error margin within ≤5%. One-way ANOVA was carried out and pair-wise comparisons were made with the control group using Dunnett’s test to maintain an acceptable error margin of 5%. A two-tailed *P* value of <0·05 was considered statistically significant.

## Result

### 1. Acute effect of aqueous fraction of *B. vulgaris* on glucose homeostasis and insulin secretion (OGTT Studies)

**Figure 1:**
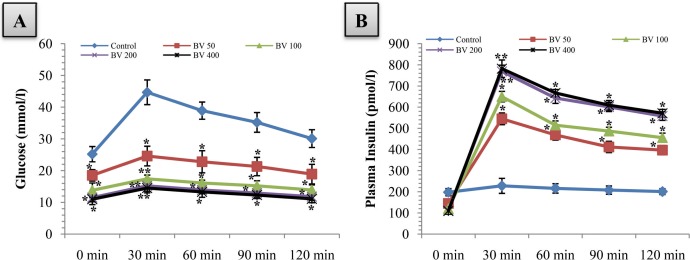
Effects of aqueous fraction of *Beta vulgaris* (BV) on A) blood glucose level and B) plasma insulin level during a glucose tolerance test in diabetic db/db mice. Values are means and standard deviations (SD) represented by vertical bars (n = 10). Fasted mice were orally given aqueous fraction of *B. Vulgaris* (50 mg/kg, 100 mg/kg, and 200 mg/kg body weight) or vehicle for control group(water; 10 ml/kg)with glucose (2.5 g/kg body weight). Mean values marked with an asterisk (*) or two (**) were significantly different from those of respective control group at p<0.05 and p<0.01 respectively. (Derived from repeated-measures ANOVA and adjusted using Bonferroni correction).

All three doses of *B. Vulgaris* were successful to keep the blood glucose level reduced throughout the very end of the experiment period, after the initial acute oral glucose insult (p<0.05). Maximum reduction in the glucose level was seen at the 30mins time point in *B. Vulgaris* treated groups whereas the steep rise continued in the untreated group (Fig. 1).

This fall in the plasma glucose level was completely justified by the corresponding increase in the blood insulin level, at the same time points, with the treatment of the three doses of *B. Vulgaris* (p<0.05). Maximal insulin release was seen at 30mins in all the *B. Vulgaris* treated groups (Fig. 1).

Interestingly, the reduction in glucose level and increase in insulin level in *B. Vulgaris* treated group were dose dependent. *B. Vulgaris* treated animals(200 mg/kg)gave the highest reduction in blood glucose and maximum increase in blood insulin level at 30mins (p<0.01) and at the rest of the time periods (p<0.05).

### 2. Acute effect of aqueous fraction of *B. vulgaris* on different insulin secreting or inhibiting hormones in glucose stimulated state

Among the hormones checked in our study, only Acetylcholine and GLP-1 levels were increased significantly on *B. Vulgaris* treatment (200 mg/kg), 30mins after an acute glucose load (p<0.05). Acetylcholine and GLP-1 levels saw increments of 55.91% and 45.58% respectively (Table 1). The plasma insulin increased 237.28% from the basal level (p<0.01).

**Table 1:** Effect of aqueous fraction of *Beta vulgaris* (BV) on different blood hormones of db/db diabetic mice at 30mins after an oral glucose load^1^ is given.

	Untreated^2^	BV treated^2^
Insulin (pmol/l)	228±35.4	769±29.4**
Epinephrine (nmol/l)	0.38±0.08	0.42±0.07
Norepinephrine (nmol/l)	1.31±0.18	1.23±0.21
Acetylcholine(pg/ml)	127±13.8	198±14.1*
Glucagon-like peptide-1 (GLP-1) (pmol/l)	14.7±1.8	21.4±1.5*
Gastric inhibitory polypeptide (GIP) (pmol/l)	41.8±5.4	43.7±7.2
Vasoactive intestinal peptide (VIP) (pmol/l)	7.3±0.7	7.1±1.0
Pituitary adenylate cyclase-activating peptide (PACAP) (pmol/l)	14.7±2.2	15.4±1.7
Insulin like growth factor-1 (IGF-1) (ng/ml)	88.4±9.7	93.1±10.2
Pancreatic polypeptide (pg/ml)	483.9±86.9	461.8±72.1
Somatostatin (pg/ml)	16.9±3.1	17.4±2.6

^1^ Glucose 2.5 g /kg body weight.

^2^ *B. Vulgaris* treated group was administered with 200 mg/kg dose. Untreated group received vehicle (water; 10 ml/kg) only. Data are presented as Mean±SD (n = 10). Mean values marked with an asterisk (*) or two (**) were significantly different from those of respective control groups at p<0.05 and p<0.01 respectively (Derived from repeated-measures ANOVA and adjusted using Bonferroni correction).

Other parameters: epinephrine, nor-epinephrine, GIP, VIP, PACAP, IGF-1, pancreatic polypeptide, and somatostatin were not changed significantly.

### 3. Confirmation of the role of *B. vulgaris* mediated increased acetylcholine on the enhanced insulin secretion with *B. vulgaris* treatment

**Figure 2:**
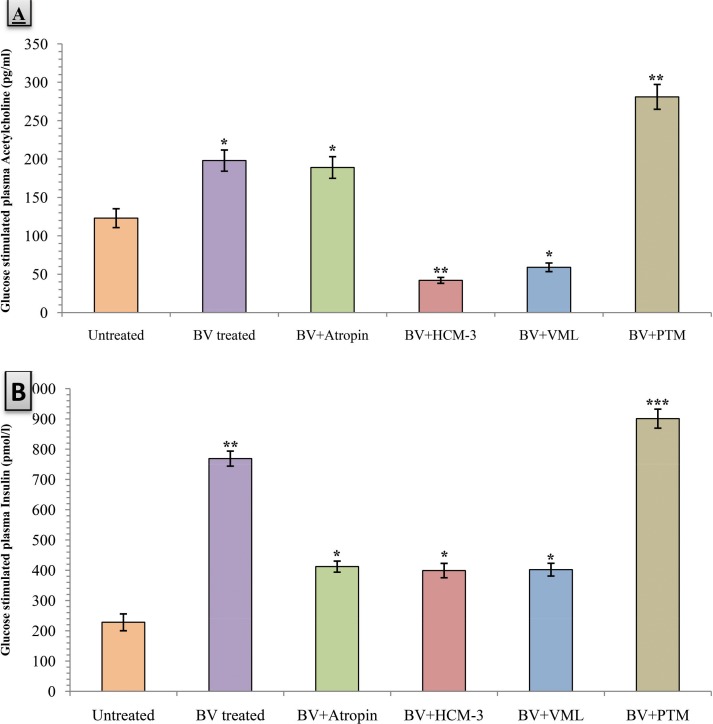
Effects of aqueous fraction of *Beta vulgaris* (BV) on A) plasma Acetylcholine level and B) associated plasma Insulin level in different treatment groups of db/db diabetic mice at 30mins after glucose load is given. Values are means and standard deviation (SD) represented by vertical bars (n = 9). Fasted mice were orally given aqueous fraction of *B. Vulgaris* (200 mg/kg) alone or with different drugs, injected intravenously 30mins before *B. Vulgaris* administration, with a glucose load (2.5 g/kg body weight). Untreated group received only vehicle (water; 10 ml/kg). Atropine: A muscarinic receptor antagonist; dose 0.1 mg/kg. HCM-3: Hemicholinium-3, a choline uptake blocker; dose 0.1 mg/kg. VML: Vesamical, an inhibitor of vesicular acetylcholine transport; 3.5 mg/kg. PTM: Physostigmine, a reversible cholinesterase inhibitor; dose 0.5 mg/kg. Mean values marked with an asterisk (*) or two (**) or three (***) were significantly higher from the control group value with p<0.05 or p<0.01 or p<0.001 respectively (Derived from One-way ANOVA followed by post hoc Dunnett’s test).

Similar to the previous experiments, *B. Vulgaris* treated group saw a concurrent increase in the levels of acetylcholine (55.91%) and insulin (237.28%)(p<0.05) (Fig. 2 A&B).

*B. Vulgaris*+Atropine treated group, also experienced an increased level of acetylcholine to a similar extent (53.66%; p<0.01) but a comparatively smaller surge in the insulin levels (80.70%; p<0.05) compared to the control, (Fig. 2 A&B).

Hemicholinium-3+*B. Vulgaris* treatment diminished the effect of *B. Vulgaris* on acetylcholine level depicted by a reduction of 65.85% than the untreated group (p<0.01). A a 75% rise in the insulin level compared to untreated group was seen (Fig. 2 A&B).

Vesamicol+*B. Vulgaris* treated group showed a reduction of 52.03% in plasma acetylcholine level compared to the untreated group (p<0.05). A 76.32% rise in plasma insulin level was seen (p<0.05) (Fig. 2 A&B).

Physostigmine+*B. Vulgaris* treated group showed a significant increase in both the acetylcholine and insulin levels (128.46% and 295.16% respectively) compared to the untreated group (p<0.01). These increases were more intense than the *B. Vulgaris* only treated group (Fig. 2 A&B).

### 4. Confirmation of the role of *B. vulgaris* mediated increased GLP-1 on the enhanced insulin secretion with *B. vulgaris* treatment

**Figure 3:**
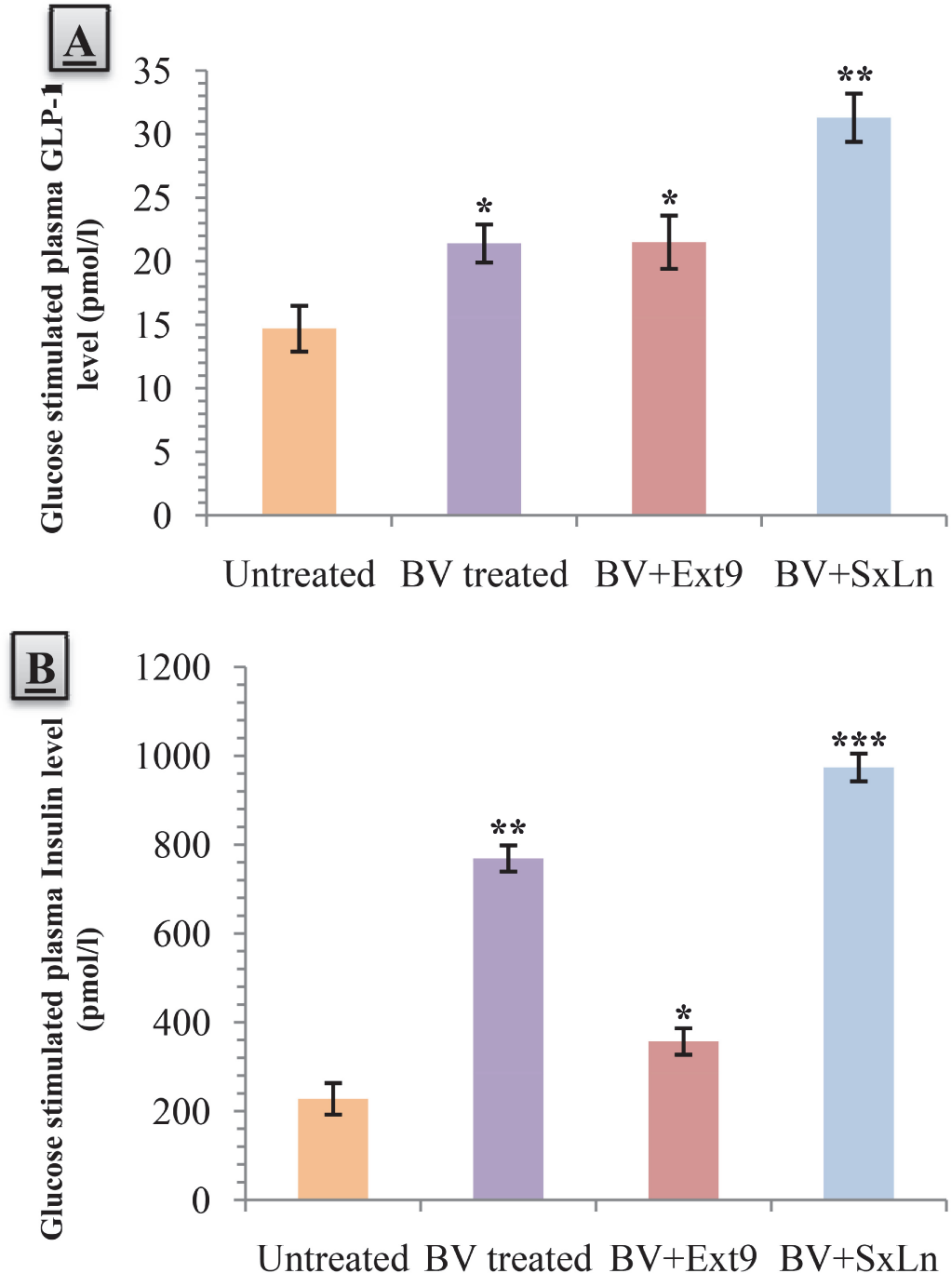
Effects of aqueous fraction of *Beta vulgaris* (BV) on A) plasma GLP 1 level and B) associated plasma Insulin level in different treatment groups of db/db diabetic mice at 30mins after glucose load is given. Values are means and standard deviation (SD) represented by vertical bars (n = 10). Fasted mice were orally given aqueous fraction of *B. Vulgaris* (200 mg/kg) alone or with different drugs along with a glucose load (2.5 g/kg body weight). Untreated group received only vehicle (water; 10 ml/kg).

*B. Vulgaris* treatment caused significant increase in plasma GLP-1 level (45.58%, p<0.05) and plasma insulin level (237.28%, p<0.01) compared to the untreated group (Fig. 3 A&B).

Ext9: Exendin (9–39), a GLP-1 receptor antagonist; dose 300 pmol/kg/min; administered through a femoral vein catheter continuously for 30mins while the animal subjects were kept under sodium pentobarbital anaesthesia. SxLn: Saxagliptin, an inhibitor of dipeptidyl peptidase-4; dose 10μmol/l; administered orally 4 h before the glucose load and *B. Vulgaris* treatment.

Mean values marked with an asterisk (*) or two (**) were significantly higher from the associated control group value with p<0.05 or p<0.01 respectively (Derived from One-way ANOVA followed by post hoc Dunnett’s test).

*B. Vulgaris*+Extendin (9–39) co-treatment gave similar, significant increase in the level of GLP-1 (46.26%, p<0.05). However, unlike GLP-1 level, the insulin level was not raised to an extent similar to the solo treatment of *B. Vulgaris*; however, the increase was significant (56.58%, p<0.05) (Fig. 3 A&B).

Saxagliptin+*B. Vulgaris* treatment caused a further increase both in the GLP-1 level (112.93%, p<0.05) and plasma insulin level (327.19%, p<0.001) (Fig. 3 A&B).

### 5. Analysis of pathway activation of *B. Vulgaris* induced enhanced Acetylcholine mediated insulin secretion

**Figure 4:**
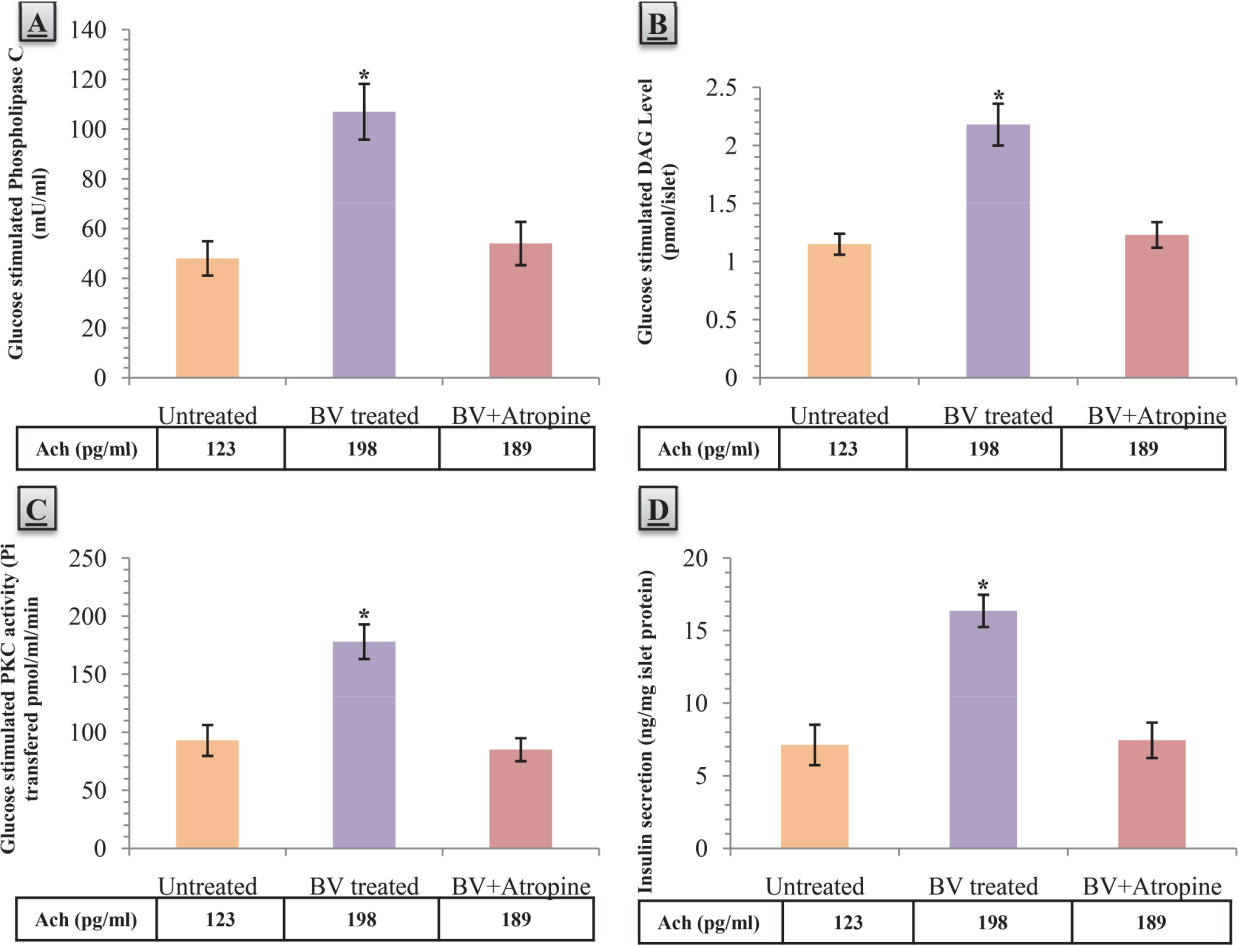
Effects of aqueous fraction of *Beta vulgaris* (BV) on A) Phospholipase C activity, B) Diacylglycerol (DAG) level, C) Protein Kinase C (PKC) activity, and D) Insulin secretion from isolated mouse islets after 30mins of incubation. Values are means and standard deviation (SD) represented by vertical bars (n = 4). Groups of 25–30 islets were isolated from db/db diabetic mice after collagenase digestion. The mice were fed with *B. Vulgaris* (200 mg/kg) for 8weeks before the isolation procedure. Isolated islets were exposed to 5.6mM for 60mins glucose to adapt to a baseline glucose concentration. Then they were exposed to 16.7mM glucose alone (untreated group), with *B. Vulgaris* (50 μg/ml) or with *B. Vulgaris* (50 μg/ml)+Atropine (1mmol/l) for 30mins. At the same time, the groups were also exposed to Acetylcholine 123 pg/ml (untreated group), 198 pg/ml (*B. Vulgaris* treated group), and 189 pg/ml (*B. Vulgaris*+Atropine treated group) (concentration of Acetylcholine found during in-vivo test in response to different treatments at 30mins). After the incubation period, samples were collected or islets were sonicated where required and equivalent amount of islet protein was analyzed. Values marked with an asterisk (*) were significantly higher from the control group value with p<0.05 (Derived from One-way ANOVA followed by post hoc Dunnett’s test). In case of PLC activity, we define 1 unit of enzyme activity as the amount that transfers 1 μmol of PI/min.

*B. Vulgaris* treated islets batch experienceda 122.92% rise in Phospholipase C (PLC) activitycompared to the control (p<0.05). Atropine+*B. Vulgaris* co-treatment abated the rise and kept the PLC activity similar to the control (Fig. 4A).

A significant rise (89.57%) in the islet DAG content was observed in the *B. Vulgaris* treated batch (p<0.05). Atropine co-treatment had a diminishing effect on *B. Vulgaris* induced DAG content (Fig. 4B).

Protein Kinase C (PKC) activity was increased by 91.40% with *B. Vulgaris* treatment (p<0.05). Atropine co-treatment, however, reduced the enhancement of PKC activity by *B. Vulgaris* (Fig. 4C).

An expected rise (129.45%) in the insulin secretion, from islets, was seen in the *B. Vulgaris* treated batchcompared to the untreated batch (p<0.05). Atropine co-treated batch failed to show any significant change in insulin secretion compared to untreated batch (Fig. 4D).

### 6. Analysis of pathway activation of *B. Vulgaris* induced enhanced GLP-1 mediated insulin secretion

**Figure 5:**
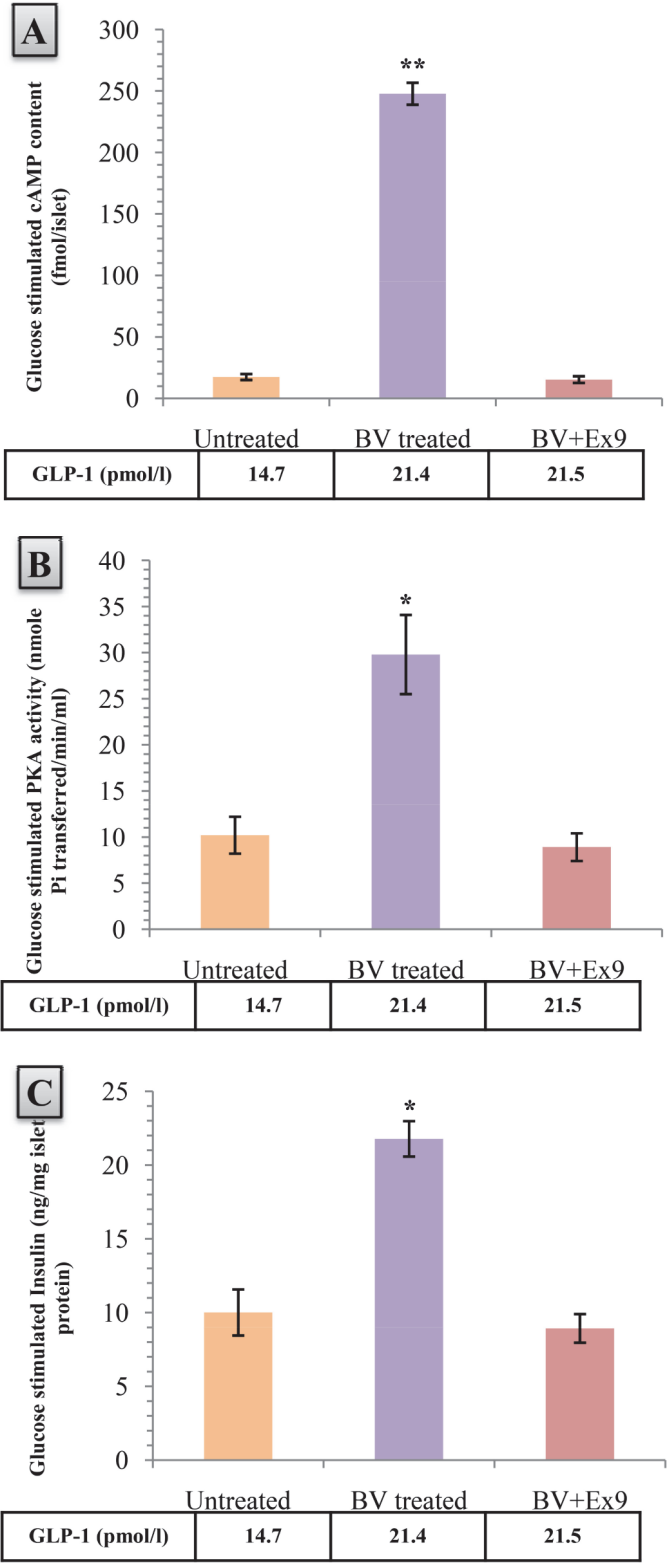
Effects of aqueous fraction of *Beta vulgaris* (BV) on A) cAMP content, B) Protein Kinase A (PKA) activity, and C) Insulin secretion from isolated mouse islets after a 30mins incubation period. Values are means and standard deviation (SD) represented by vertical bars (n = 4). Groups of 25–30 islets were isolated from db/db diabetic mice after collagenase digestion. The mice were fed with *B. Vulgaris* (200 mg/kg) for 8weeks before the isolation procedure. Isolated islets were exposed to 5.6mM glucose for 60minsto adapt to a baseline glucose concentration. Then they were exposed to 16.7mM glucose alone (untreated group) or to *B. Vulgaris* (50 μg/ml) or to *B. Vulgaris* (50 μg/ml)+Ext9 (Extendin 9–39) (2μmol/l) for 30mins. At the same time, the groups were also exposed to GLP-1 14.7pmol/l (untreated group), 21.4 pmol/l (*B. Vulgaris* treated group), and 21.5pmol/l (*B. Vulgaris*+Extending 9–39 treated group) (concentrations of GLP-1 found during in vivo test in response to different treatments at 30mins). After the incubation period, samples were collected or islets were sonicated, where required, and equivalent amount of islet protein was analyzed. Values marked with an asterisk (*) or two (**) were significantly higher from the control group value with p<0.05 or p<0.01 respectively (Derived from One-way ANOVA followed by post hoc Dunnett’s test).

*B. Vulgaris* treatment managed to produce a1324.18% increase in the cAMP content of the respective islet batch (p<0.01). But co-treatment with Extendin (9–39) abolished the increase (Fig. 5A).

An increase of 192.17% in the Protein Kinase A (PKA) activity was observed in the *B. Vulgaris* treated islet batch (p<0.05). Extendin (9–39)+*B. Vulgaris* co-treated showed no significant difference in activity (Fig. 5B).

Insulin secretion from the islet batch treated with *B. Vulgaris* was increased compared to the untreated batch by 152.6% (p<0.05). In *B. Vulgaris*+Extendin (9–39) co-treated islets, a rise in insulin levels were seen however, the increase was not significant (Fig. 5C).

### 7. Effects of *B. vulgaris* on GLUT1 and GLUT4 transporter content in membranes of skeletal myocytes

**Figure 6:**
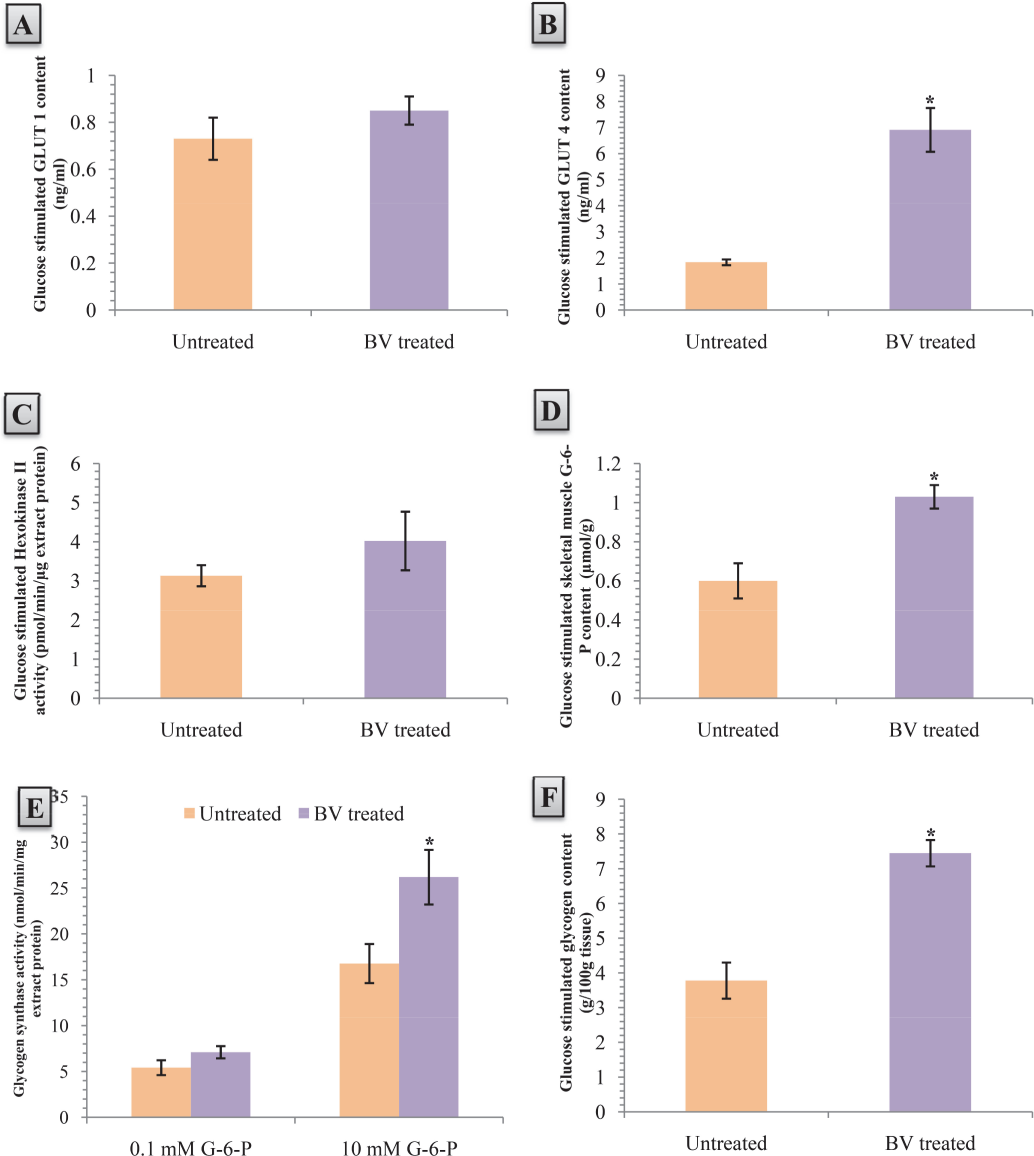
Effects of aqueous fraction of *Beta vulgaris* (BV) on A) GLUT 1 level & B) GLUT 4 level in skeletal smooth muscle membrane, C) Hexokinase II activity, D) Glucose-6-Phosphate (g-6-P) content, E) Glycogen synthase activity, and F) Glycogen content in skeletal smooth muscle in different treatment groups of db/db diabetic mice at 1h after glucose load is given. Values are means and standard deviation (SD) represented by vertical bars (n = 8). Fasted mice were orally given aqueous fraction of *B. Vulgaris* (200 mg/kg) or vehicle (water; 10 ml/kg) along with a glucose load (2.5 g/kg body weight) 1h before the smooth muscle membrane and smooth muscle was collected and processed as described in the material and method section for analysis. Mean values marked with an asterisk (*) were significantly higher from the associated control group value with p<0.05 (Derived from Student’s t-test).

The amount of membrane docked GLUT1 transporter was not altered significantly in the skeletal myocytes of the *B. Vulgaris* treated group (Fig. 6A). Interestingly, GLUT4 amount massively increased in the *B. Vulgaris* treated group compared to the untreated group(277.60%, p<0.05) (Fig. 6B).

### 8. Effects of *B. vulgaris* on Hexokinase II activity, Glucose-6-phosphate content, and Glycogen synthase activity in skeletal myocytes

Hexokinase II activity increased in the skeletal myocytes of *B. Vulgaris* treated group; however it was not significant (Fig. 6C).

Glucose-6-phosphate content was significantly raised by 71.67% in the *B. Vulgaris* treated group compared to the untreated group after the 8weeks treatment period (p<0.05) (Fig. 6D).

Significantly enhanced glycogen synthase activity was found in the *B. Vulgaris* treated group, after the 8weeks treatment period, when had got stimulated by 10mM G-6-P (56.23%, p<0.05). However, no significant change was observed in the presence of 0.1mM G-6-P (Fig. 6E).

### 9. Effects of *B. vulgaris* on muscular glycogen content

The 8weeks treatment with *B. Vulgaris* resulted in significantly increased amount of muscular glycogen compared to the untreated group (97.09%, p<0.05) (Fig. 6F).

## Discussion

Insulin secretion may occur due to glucose or non glucose stimuli. The fuel stimuli being glucose, free fatty acids, triglycerides etc while the non fuel stimuli being hormones such as acetylcholine, GLP-1, epinephrine etc [14,19–23]. The objective of the current study was to further explore the probable anti-hyperglycemic of action of *B. vulgaris* and to expose its underlying molecular mechanisms. The oral glucose tolerance test (OGTT) performed in diabetic mice provided us with a concrete foundation for our subsequent studies. It was observed that *B. Vulgaris* treated mice were more tolerant to oral glucose challenges, demonstrating anti-hyperglycemic activity of the plant extract. Our study corroborates previous findings, which too demonstrated a successful amelioration of diabetic hyperglycemia[1,4]. It is noteworthy that no other fractions but the aqueous *B. Vulgaris* fraction showed significant activity.The aqueous fraction was found active ata much lower dose of 50mg/kg.Previous studied werewere mostly conducted at a much higher dose of 2000mg/kg. Therefore, we may conclude that the aqueous fraction of *B. Vulgaris* probably holds the active constituents responsible for the anti-hyperglycemic activity and could be further analyzed to discover the bioactive molecules. However, none of the above studies attempted to conduct a mechanistic study on their preliminary observations. Researchers have clearly shown a marked co-relation between diabetic hyperglycemia and various micro and macro vascular complication, retinopathy, neuropathy, nephropathy, hypertension, oxidative stress etc [24–29]. Investigators have found that hyperglycemic patients admitted in hospitals with general health complication or special pathological conditions like stroke or myocardial infarction have a higher mortality rates [30]. It is therefore, of a great clinical significance to maintain the plasma glucose concentration near the baseline level[31]. While numerous anti-hyperglycemic agents exist to control the glycemic spikes diabetic patients experience, they are expensive, usually requires a lifetime treatment regimen and are poorly integrated with the patients lifestyle [32–34]. As a result, perhaps, diabetic nutrition therapy has found its rightful place in diabetic management [2,3,35].

The current study adds a newer dimension in glycemic study of *B. vulgaris* in the respect that most previous investigators have employed Type 1 streptozotocin or alloxan induced diabetic model [1,4], which had a deleterious effect on the pancreas [36]. The previously used models, consequently, rendered us unable to observe any possible insulinotropic activity *B. vulgaris* treatment might have in Type 2 diabetic conditions. During the oral glucose tolerance test, we observed that the plasma insulin level rose significantly higher in the *B. vulgaris* treated mice as opposed to the control group. We may, therefore, partly implicate the observed anti-hyperglycemic effect to the insulin secretagogue activity of *B. vulgaris*. Increased plasma insulin levels, can correct hyperglycemia not only in Type 1 but also in Type 2 diabetic patients [37]. The current findings therefore impart an important clinical significance on Type 2 diabetic patients as well. The main objective of the present study, however, was to demonstrate a probable mechanism of insulin secretion by *B. vulgaris* administration.

In order to screen out any possible hormone that might be responsible for insulin secretion. We assayed the plasma levels of Epinephrine, Noreinephrine, Acetylcholine, GIP, VIP, GLP-1, PACAP, IGF-1, PP and Somatostatinin conjunction with the corresponding in-vivo plasma insulin levels. In the initial screening tests, we found a marked rise in plasma Acetylcholine and GLP-1 levels along with the concurrent rise in plasma insulin level in the *B. vulgaris* treated group. The insulin level reached its maximum at 30mins after the final administration of the extract. The plasma levels of the other hormones remained largely unchanged across groups, and was therefore excluded from the study beyond this point. Investigators have previously demonstrated that a rise in plasma Acetylcholine or GLP-1 level stimulates enhanced insulin secretion [38–41]. Acetylcholine is known to initiate a major molecular cascade in the insulin secretory pathway [42]. GLP-1, on the other hand, is being actively developed as a potential drug for diabetic patients [43–45]. Our preliminary findings, therefore, support the established positive correlation between insulin with acetylcholine and GLP-1. It also provides us with the first glimpse of the mechanism through which *B. vulgaris* might initiate this insulin release. To further validate the role of Acetylcholine and GLP-1 in insulin secretion, we treated groups of test animals with inhibitors,antagonists, or enhancers of these two endogenous substances. Four separate *B. Vulgaris* treated groups were administered Atropine (an Acetylcholine receptor antagonist), Hemicholinium-3 (choline uptake inhibitor), Vesamicol (inhibitor of Vescicular Acetylcholine transport) and Physostigmine (inhibitor of Acetylcholinesterase). Hemicholinium and Vesamicol lowered the acetylcholine levels in the *B. vulgaris* treated group, while Physostigmine administration increased the plasma acetylcholine levels even higher than the group receiving *B. vulgaris* only. Mice receiving Hemicholinium and Vesamicol along with *B. Vulgaris* experienced a consequential reduction in both acetylcholine and insulin levels. Physostigmine administered mice experienced a four fold increase in plasma insulin, the highest amongst all the groups, which can be attributed to the maximum level of plasma acetylcholine. Mice administered with atropine, regardless showing a high plasma acetylcholine, did not demonstrate a rise in plasma insulin, probably, due to a blockage of the cholinergic receptors responsible for activation of the insulin secreting pathway. This showed *B. Vulgaris* increased Acetylcholine which in turn increased level of plasma insulin.

GLP-1, an insulinotropic hormone also demonstrated a rise in our preliminary screening, hence, was considered to be our second candidate for further analysis. Groups of mice were co-administered *B. vulgaris* along with Saxagliptin (selective inhibitor of GLP-1 degrading enzyme DPP4) and Extendin (9–39) (GLP-1 receptor antagonist). Saxagliptin reduced GLP-1 degradation in the plasma and thus increased the plasma GLP-1 level while Extendin antagonized GLP-1 activity. After co-administration of Saxagliptin and *B. vulgaris*, the GLP-1 level was elevated, the most,This in turn increased the plasma insulin levels. On the other hand, *B. vulgaris* co-administered with Extendin, while retained the elevated plasma GLP-1 level, showed a marked reduction in insulin secretion, allowing us to conclude that GLP-1 receptor antagonism might be responsible for the observed reduction. On *B. vulgaris* treatment alone, the diabetic test animals experienced an approximately three fold increase in plasma insulin levels.

When *B. vulgaris* was singly administered to the diabetic mice, it resulted in a greater than3 fold increase in the plasma insulin level. Taking either GLP-1 or Acetylcholine out of the equation using the aforementioned antagonists and inhibitors reduced the insulin release to only 1.5 times that of the baseline level. Half the amount of the initially observed insulin was still being secreted, in either the blockage of GLP-1 or Acetylcholine. From these results we may surmise that the role of both these hormones were roughly equal in *B. vulgaris* mediated insulin secretion.

In our isolated islet studies, we incubated the islets in the nutrient medium along with Acetylcholine and GLP-1 at the same concentrations as observed in-vivo in the *B. vulgaris* treated and control groups. The objective of these experiments were to ensure whether the observed plasma levels of Acetylcholine and GLP-1 were capable of activating the insulin secreting cascade reactions. Separate identical islet batches were prepared, with Atropine (cholinergic receptor antagonist) and Extendin (GLP-1 receptor antagonist) supplemented nutrient media as negative control groups. Islets incubated in the physiological acetylcholine concentrations of *B. vulgaris* treated mice showed a significant rise in PLC, PKC-α activity and DAG levels, along with increased insulin release after 30mins of incubation. Our results, therefore, concludes that the acetylcholine induced pathway for insulin release, as suggested by Prentki et al and Zawawalich et was activated by the observed increase in physiological acetylcholine with *B. vulgaris* treatment[14]. On the contrary, in the control and negative control (blank and atropine supplemented) batches, these signaling molecules remained at their basal levels. These results unequivocally confirmed that the observed physiological levels of acetylcholine after *B. vulgaris* treatment are indeed capable of initiating the previously proposed, acetylcholine mediated, insulin secreting pathway.

The primary effector of GLP-1 induced insulin secretion is cAMP, and cAMP mediates its insulinotropic effect via the PKA-dependent phosphorylation of downstream targets[15]. Therefore, increased cAMP levels and PKA activity on exposure to physiological levels (in *B. vulgaris* treated group) of GLP-1 can be held indicative to activation of GLP-1 mediated insulin secreting pathway. The control and negative control batches were found to possess basal PKA activity, cAMP and insulin levels. While the batch incubated with GLP-1 only, showed significantly increased levels of the aforementioned molecules. Since the concentration of GLP-1 used in the islet experiments was the same as observed in the *B. vulgaris* treated animals we may conclude that this concentration is sufficient for activating the GLP-1 mediated insulin secreting pathway. Increased GLP-1 might also impart the added effect of reducing apoptosis and increasing the proliferation in pancreatic β-cells as previously established by Wang et al. The study conducted on INS-1 cell line has identified this effect to be mediated by Protein Kinase B [46]. While it is not certain whether Wang’s findings, on INS-1 insulinoma cell lines, will hold true in our in-vivo model, his findings leave us wonder whether such observations can be replicated in-vivo as well. Nevertheless, further studies are needed to conduct a detailed histological examination of the mice pancreas to observe the cell morphology and relative propensity to undergo apoptosis or proliferation in the above.

Increased peripheral glucose uptake in the liver and skeletal myocytes are key targets for managing post-prandial glycemic spikes, especially in Type 2 diabetes mellitus[47]. In the current study, we chose to observe the glucose disposal in the skeletal myocytes because most of the glucose are dumped in myocytes and the primary route of glucose entry in these cells are the GLUT1 and GLUT4 transporters[48–52]. Investigators have also found that levels of glucose transporter proteins show a strong correlation with insulin stimulated glucose disposal in humans[53]. In the present study, we quantified the membrane docked GLUT1 and GLUT4 transporters due to their particular abundance in the skeletal muscle cells. This was subsequently followed by assaying the Hexokinase II activity, glucose-6-phosphate levels, Glycogen synthase activity, and glycogen content in homogenized muscle samples. Results from the our study showed an elevation in membrane bound GLUT4 transporter levels, while GLUT1 levels remained unaltered after B.vulgaris treatment. Increased levels of membrane bound GLUT4 transporters explains the elevated glucose-6-phosphate amount in the muscle cells, with the muscle Hexokinase II activity remaining at the baseline level. This leads to us to conclude that the increased GLUT4 transporter is the predominantly responsible transporter for most of the elevated the glucose uptake. Glucose entering the skeletal myocytes are rapidly phosphorylated to glucose-6-phosphate which is an essential raw material for glycogen synthesis. It also helps to increase activity of the enzyme Glycogen synthase by allosterically binding with it[54]. Hence, a rise in its level can be viewed as a harbinger to increased glycogen formation in the muscle. Subsequent assays showed a rise in muscular glycogen levels which can be logically linked to the enhanced activity of the enzyme glycogen synthase, hence, validating our initial conjecture. The current study, however, is limited in determining the details of insulin signaling by *B. vulgaris* on Insulin Receptor phosphorylation, Insulin Receptor Substrate Phosphorylation, Protein Kinase B and C activation in the target tissues. *B. vulgaris* might be capable of altering any of the above mentioned parameters, thereby greatly capable of increasing the insulin sensitivity of the target organs, namely, liver and skeletal muscles. Further studies would be invaluable in determining how or whether the enhanced insulin secretion is improving the body’s overall sensitivity to insulin.

## Conclusion

The major conclusions that can be derived from this study are as follows:

1. Aqueous fraction of the ethanol extract of *B. Vulgaris* is the only active fraction and has potent anti-hyperglycemic activity (active at as low as 50mg/kg).
2. The anti-hyperglycemic activity of *B. vulgaris* is partly insulin mediated and partially due to the enhanced glucose disposal in the skeletal myocytes.
3. *B. vulgaris* induced enhancement of glucose stimulated insulin secretion is mediated by a rise in plasma Acetylcholine level and GLP-1 level. In-vivo concentrations of Acetylcholine and GLP-1 are capable of initiating the insulin secreting cascade reaction in-vitro.
4. *B. vulgaris* induced enhanced glucose disposal in the skeletal myocytes is due to increased number of plasma membrane docked GLUT4 transporters. The glucose taken up is rapidly converted to glycogen due to increased activity of glycogen synthase in *B. vulgaris* treated mice.

## Supporting Information

S1 Table Effect of different fractions of the ethanol extract of *Beta vulgaris* (BV) on blood glucose level and plasma insulin level of db/db diabetic mice at 30 minutes after an oral glucose load is given. (DOCX)
