# Supplementary material for: Aqueous Fraction of Beta vulgaris Ameliorates Hyperglycemia in Diabetic Mice due to Enhanced Glucose Stimulated Insulin Secretion, Mediated by Acetylcholine and GLP-1, and Elevated Glucose Uptake via Increased Membrane Bound GLUT4 Transporters
Source: PLoS One. 2015 Feb 3;10(2):e0116546. doi: 10.1371/journal.pone.0116546 (PMC4315578; doi:10.1371/journal.pone.0116546)
Supplement: S1 Table — (DOCX) [file pone.0116546.s001.docx]

| Table 1: Effect of different fractions of the ethanol extract of *Beta vulgaris* (BV) on blood glucose level and plasma insulin level of db/db diabetic mice at 30 minutes after an oral glucose load^1^ is given. | | |
| --- | --- | --- |
|  | Blood glucose (mmol/l) | Plasma Insulin (pmol/l) |
| Untreated | 25.8±5.8 | 201.3±18.1 |
| Aqueous fraction^2^ | 11.1±2.1** | 783±23.7** |
| n-Hexane fraction^2^ | 23.6±4.9 | 208±15.1 |
| Chloroform fraction^2^ | 22.1±6.2 | 202±11.9 |
| Ethyl acetate fraction^2^ | 27.9±7.2 | 196.8±19.2 |
| 1-Butanol fraction^2^ | 19.6±5.1 | 213±21.7 |
| ^1^ _Glucose 2.5 g /kg body weight._  ^2^ _Treatment groups were administered with 400 mg/kg dose. Untreated group received vehicle (water; 10 ml/kg) only._  _Data are presented as Mean±SD (n= 13). Mean values marked with an asterisk (*) or two (**) were significantly different from those of respective control groups at p<0.05 and p<0.01 respectively (Derived from repeated-measures ANOVA and adjusted using Bonferroni correction)._ | | |
